# Instance Segmentation and Berry Counting of Table Grape before Thinning Based on AS-SwinT

**DOI:** 10.34133/plantphenomics.0085

**Published:** 2023-08-29

**Authors:** Wensheng Du, Ping Liu

**Affiliations:** ^1^ Shandong Agricultural Equipment Intelligent Engineering Laboratory; Shandong Provincial Key Laboratory of Horticultural, Machinery and Equipment; College of Mechanical and Electronic Engineering, Shandong Agricultural University, Tai’an 271000, China.; ^2^ School of Construction Machinery, Shandong Jiaotong University, Jinan 250357, China.

## Abstract

Berry thinning is one of the most important tasks in the management of high-quality table grapes. Farmers often thin the berries per cluster to a standard number by counting. With an aging population, it is hard to find adequate skilled farmers to work during thinning season. It is urgent to design an intelligent berry-thinning machine to avoid exhaustive repetitive labor. A machine vision system that can determine the number of berries removed and locate the berries removed is a challenge for the thinning machine. A method for instance segmentation of berries and berry counting in a single bunch is proposed based on AS-SwinT. In AS-SwinT, Swin Transformer is performed as the backbone to extract the rich characteristics of grape berries. An adaptive feature fusion is introduced to the neck network to sufficiently preserve the underlying features and enhance the detection of small berries. The size of berries in the dataset is statistically analyzed to optimize the anchor scale, and Soft-NMS is used to filter the candidate frames to reduce the missed detection of densely shaded berries. Finally, the proposed method could achieve 65.7 *AP^box^*, 95.0 $AP_{0.5}^{\mathrm{box}}$, 57 $AP_{s}^{\mathrm{box}}$, 62.8 *AP*^*mask*^, 94.3 $AP_{0.5}^{\text{mask}}$, 48 $AP_{s}^{\text{mask}}$, which is markedly superior to Mask R-CNN, Mask Scoring R-CNN, and Cascade Mask R-CNN. Linear regressions between predicted numbers and actual numbers are also developed to verify the precision of the proposed model. *RMSE* and *R*^2^ values are 7.13 and 0.95, respectively, which are substantially higher than other models, showing the advantage of the AS-SwinT model in berry counting estimation.

## Introduction

With the development of table grape cultivation, the demand for high-quality grapes is gradually increasing. High-quality grapes with beautiful bunch shape, bright colors, and uniform berry size often require more precise management [1–3]. Berry thinning is a critical process in management, because by thinning, the bunches have a compact and well-balanced shape, and each berry has sufficient space to grow without interfering with others [4]. Currently, the best time to thin berries is within 2 weeks and requires a large number of skilled farmers. It is time-consuming and laborious to thin the berries. For some large-scale vineyards, because it is more difficult to find a sufficient number of skilled farmers during the thinning season, the best time for berry thinning is often missed. It is crucial to design a berry-thinning machine to meet the needs of table grape management. Therefore, it could not only improve the efficiency of management but also benefit managers.

In order to achieve high-quality grapes through a thinning machine, a relevant vision system for thinning detection and segmentation is one of the bottlenecks to the machine. Related scholars have done some research. Vision system includes grape inflorescence detection and grape berry detection. For grape inflorescence thinning, Du et al. [5,6] proposed an algorithm to locate the grape stem clamping point and an algorithm to detect the spikelets to be removed, respectively. Buayai [7] designed smart glasses based on a deep learning (DL) algorithm to estimate the length of remaining inflorescence. In addition, the smart glasses could be configured with different DL algorithms for berry counting and berry removal identification to assist inexperienced farmers with bunch thinning [8]. For a vision system of berry thinning, it is necessary to determine the number of berries per bunch before detecting the berries to be removed. Because the removal number of berries is often different for each bunch, the number of berries after inflorescence thinning is different. What is more, the number of berries in boutique grapes is within a certain range, for example, 60 to 70 per bunch for boutique “Shine Muscat” grown in China. It is important to accurately count the number of berries in a single bunch before detecting their removal.

Research on grape berry counting can be categorized into 2 approaches: one focuses on analyzing whole vine images, while the other utilizes single-grape-bunch images. Within the first approach, some researchers have employed a combination of traditional image processing methods and machine learning techniques to detect and count grape berries [9–11], while traditional image processing methods mainly rely on manually designed feature extractors, which have poor generalization ability and robustness. With the development of computer technology, DL [12] has been applied in the field of agricultural engineering. DL with obvious advantages in accuracy, speed, and robustness has attracted much attention from academia and are widely used in the vision system in recent years. For berry counting, some approaches have been carried out on detection and segmentation of bunches by DL. Palacios et al. [13] developed a method to segment single berry, which could be used by a machine learning regression model built to estimate the yield of actual berries per vine. Coviello et al. [14] proposed GBCNet to count berries for accurate yield estimation. Zabawa et al. [15] used a convolutional neural network (CNN) and connected component algorithm to counting berries. Deng et al. [16] proposed a 2-stage pipeline TSGYE to efficiently count berries. These methods are employed to count grape berries in natural environments with robustness thanks to DL. While these methods count all the berries on the grape bunches in the whole image without distinguishing each grape bunch, it is difficult to accurately count the number of grape berries in a single bunch during thinning.

For the approaches of using single-grape-bunch images, Aquino et al. [17] used 6 descriptors as input to the classifier and compared the results of 2 classifiers to detect single bunches of grape berries. Based on this method, the research also developed a smartphone application for assessing the berries number in cluster. Liu et al. [18] developed a smartphone application to estimate the number of berries in a single grape bunch using image processing and 3D reconstruction. Luo et al. [19] proposed a method based on image processing and geometric morphology to detect berries in a single bunch and counting berries. However, the above researches are based on the traditional image processing method detection in artificial background. The experimental conditions are relatively ideal, and these methods could still have defects in traditional image processing methods, lacking robustness and generalization. To meet these challenges, Buayai et al. [8] detected grape berries from single-bunch images using deep neural networks combined with 6 machine learning regression methods for estimating the number of actual berries.

Although DL algorithms perform well for berries in a single bunch, it still has the potential for improvement in detection accuracy. Moreover, the grape berries are very small in the thinning season, which greatly limits the detection accuracy of grape berries. In recent years, counting small fruits has posed challenges due to their small size and low resolution. Scholars have proposed various detection methods for small fruits such as apples [20], cherry fruits [21], tomatoes [22], and passion fruits [23]. These methods demonstrate encouraging detection accuracy and speed for these small fruits. It is a challenge to detect the small grape berries because the color of small grape berries is similar to that of the surrounding leaves, and grape berries are densely packed and shaded by each other in a single bunch. Therefore, an algorithm named AS-SwinT is developed to detect these small berries to improve the overall detection accuracy of the single bunch.

In this study, the main contributions of this study are as follows:

1. Adaptively spatial feature fusion (ASFF) is fused into AS-SwinT to synthesize detail and semantic and scale features. Through multilevel fusion, ASFF can extract rich detail information of small grape berries. In addition, the degree of fusion of features is dynamically adjusted by the adaptive weights of ASFF, so that the model pays more attention to the region where the small fruit berries are located, which improves the detection of small grape berries.

2. The combined use of Soft-NMS and anchor scales optimization in region proposal network (RPN) further improves the detection performance of grape berries. Soft-NMS can retain the occluded grape berries and reduce the false detection rate. In addition, the anchor scales optimization can better adapt to the size of small berries, reduce the scale error, and improve the localization accuracy of small berries.

3. Ablation experiments are conducted to validate the importance of different components in AS-SwinT and to further explain how these components interact with each other for efficient detection and segmentation. Comparison experiments with Mask R-CNN, Mask Scoring R-CNN, and Cascade Mask R-CNN are also conducted to validate the performance of AS-SwinT for the detection and segmentation of grape berries.

4. A linear regression model is developed to predict the number of berries in a single bunch of grapes more accurately. Comparing the linear fitting accuracy of the 4 models, the results show that the counting accuracy of AS-SwinT is substantially higher than the other models.

The paper is structured as follows. Materials and Methods is materials and methods that introduce the datasets and overview architecture of our model and each component used in this study. Results contains evaluation metrics, experiments setting, ablation studies, results analysis, and visualization. Discussion provides discussion, future work, and conclusion of this paper.

## Materials and Methods

### Image acquisition and labeling

The images of grapes were captured from Shandong Agricultural University Horticultural Experiment Station in Tai’an Shandong Province, where grapes were grown in a standard trellis style on 2021 May 28. The shooting device is an iPhone SE phone with a resolution of 3,024×4,032. After filtering, there were a total of 717 images, including 501 images for training dataset, 144 images for validation dataset, and 72 images for test dataset. For a comparison and transfer learning of the model in different environments, images in artificial backgrounds are captured as another dataset as shown in Table S1. The captured images were scaled to 1,024×1,365 for reducing the loading and calculation time of images, and each berry of one bunch in the captured images was labeled using the professional labeling software Labelme. The captured images with different backgrounds are shown in Fig. S1.

### Proposed methodology

**Fig. 1.**
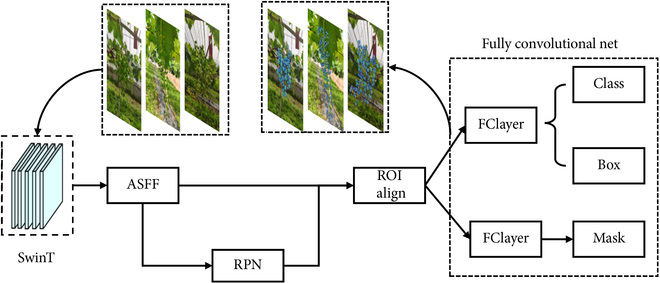
The structure of AS-SwinT.

Instance segmentation, one of the challenging tasks in machine vision, requires the generation of pixel-level segmentation masks for each object on the basis of image classification [24]. Different from semantic segmentation, instance segmentation needs to distinguish different instances of the same class. Instance segmentation is represented by Mask R-CNN [25] as a 2-stage algorithm that usually detects first and then segments. Inspired by this, we proposed the AS-SwinT model as shown in Fig. 1. The model uses Swin Transformer-Tiny (SwinT-T) [26] as the backbone network to extract features and performs feature fusion in ASFF [27], combined with RPN [28] to extract regions of interest, and completes the detection and segmentation tasks in a fully convolutional network, respectively.

#### Architecture of SwinT-T

Recently, Transformer has achieved the state of the art in natural language processing results. Inspired by this, Transformer used in computer vision such as classification, object detection, and segmentation has made impressive achievements, while SwinT is a Transformer model with sliding windows and multi-scale features. Due to its impressive performance, the Swin Transformer has been introduced to intelligent agriculture. Guo et al. [29] presented a method called Convolutional Swin Transformer based on SwinT to detect the degree and kind of plant diseases. Wang et al. [30] et al. proposed a backbone based on SwinT to recognize the practical cucumber leaf diseases. Bi et al. [31] used SwinT to improve maize seed recognition.

**Fig. 2.**
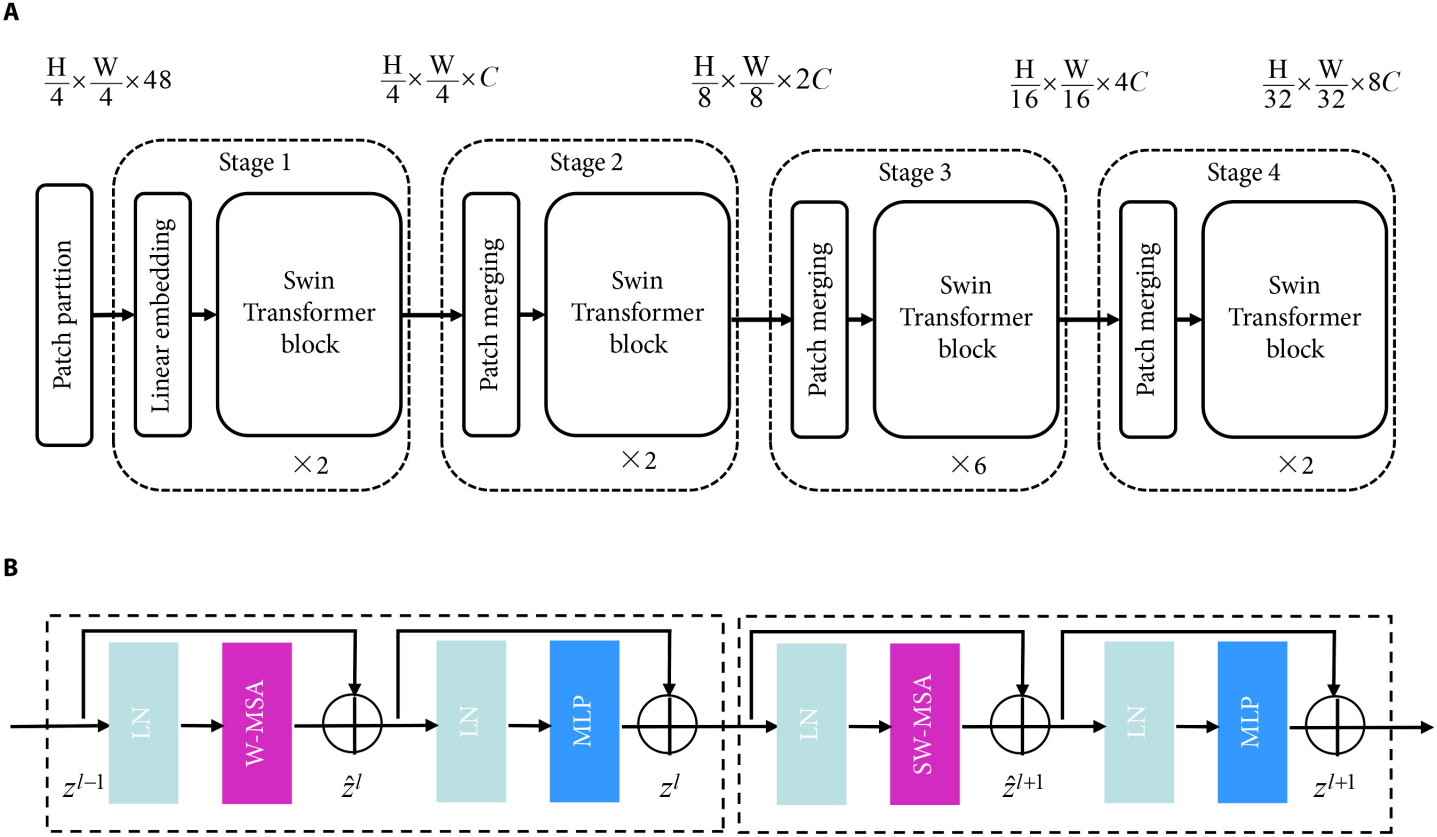
Swin Transformer architecture. (A) The structure of Swin Transformer. (B) The Swin Transformer blocks.

Figure 2 shows the architecture of SwinT-T, and it adopts a hierarchical construction that not only has the advantage of CNN to processing large-size images but also uses sliding window to establish long-range dependencies, which reduce the amount of calculation. The Swin Transformer contains 4 stages similar to ResNet [32], and each stage is fully similar repetitive units. Similar to Transformer, the image with a resolution of H×*W*×C is split into 4×4 by the Patch partition layer to get the feature H/4×W/4×48. In Stage 1, the feature dimension of the patches is changed to C by linear embedding, and then the feature is sent to Swin Transformer block. In Stages 2 to 4, there are one patch merging and several Swin Transformer blocks. The resolution of feature map in each stage is reduced to half. With the SwinT model, 4 different scales of feature maps are obtained. Swin Transformer Block includes LayerNorm (LN), Windowed Multi-head Self-attention (W-MSA), and Multilayer Perceptron (MLP). The block is expressed as:

$$\begin{array}{c} {\hat{Z}}^{l}=W-\mathrm{MSA}\left(LN\left(Z^{l-1}\right)\right)+Z^{l-1}\\ Z^{l}=\mathrm{MLP}\left(LN\left({\hat{Z}}^{l}\right)\right)+{\hat{Z}}^{l}\\ {\hat{Z}}^{l+1}=\mathrm{SW}-\mathrm{MSA}\left(\mathrm{LN}\left(Z^{l}\right)\right)+Z^{l}\\ Z^{l+1}=\mathrm{MLP}\left(\mathrm{LN}\left({\hat{Z}}^{l+1}\right)\right)+{\hat{Z}}^{l+1} \end{array}$$ (1)

where *Z^l^* is the output feature of MLP and ${\hat{Z}}^{l}$ is the output feature of W-MSA or SW-MSA. Compared with MSA used in ViT, W-MSA module is able to reduce the amount of computation by performing self-attentive computation for the inner part of the divided window. SW-MSA is introduced to enable the interaction of information between different windows and to obtain more global and accurate information. It could complete the pixel self-conscious calculation of offset windows, which indirectly increases the network perception field and improves the efficiency of information utilization.

#### Architecture of ASFF

**Fig. 3.**
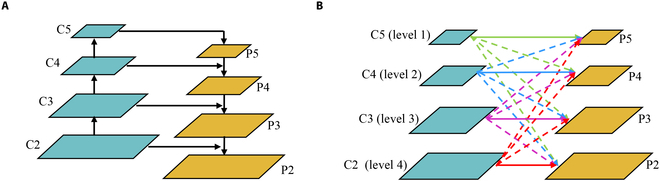
Comparison of neck network. (A) The structure of FPN. (B) The structure of ASFF in AS-SwinT.

Feature maps at different scales contain different levels of semantic information, and by fusing multiscale features, the instance segmentation could get more accurate prediction results. Feature pyramid network (FPN) [33] shown in Fig. 3A is commonly used to obtain more rich feature information in instance segmentation. However, inconsistency between different feature scales is the main limitation of FPN. In order to fuse features of grape berries under different receptive fields, ASFF is adopted, which could ensure the model to make use of feature information at different scales. The original ASFF was developed based on YOLOV3 to learn the connections among 3 different scale feature maps. The algorithm allows the network to learn the weights for each position on every feature layer, enabling it to adaptively fuse features with important information. Before fusion, all feature layers are converted to the same resolution and then trained to determine the optimal weights. To adapt SwinT-T, ASFF was extended to connections of 4 different scale feature maps as shown in Fig. 3B. The scale of the feature maps C2-C5 by SwinT-T is inconsistent, and the up-sampling or down-sampling of different levels adjusts the scale to the same size to obtain the feature maps. $X_{\mathrm{ij}}^{1\to l}$, $X_{\mathrm{ij}}^{2\to l}$, $X_{\mathrm{ij}}^{3\to l}$, $X_{\mathrm{ij}}^{4\to l}$ denotes the feature vector at the position (*i*, *j*) on the feature maps resized from levels 1, 2, 3, 4 to level *l* (*l* ∈ {1, 2, 3, 4}). For feature maps of the same size with different channels, only a 1×1×256 convolutional layer is employed to adjust the number of channels. For up-sampling, a 1 × 1 convolutional layer is applied to adjust channels to 256 dimensions, and then the feature map is up-sampled by a 2×, 4×, or 2× followed by 4× interpolation operation to reach a 2×, 4×, or 8× size. For the downsampling with 1/2 ratio, we use a 3 × 3 × 256 convolutional layer to adjust the feature map by half the size. For the downsampling with 1/4 ratio, we perform the maximum pooling operation before the convolutional layer. For the downsampling with 1/8 ratio, 2 maximum pooling operations are carried on before the convolutional layer. At each level, 4 resized feature maps with a same channel number of 256 are acquired to fuse. Feature fusion is to assign 4 learnable weights to these 4 feature maps and add them together. The feature fusion mechanism at level *l* is as follows:

$$Y_{\mathrm{ij}}^{l}=\alpha_{\mathrm{ij}}^{l}X_{i,j}^{1\to l}+\beta_{\mathrm{ij}}^{l}X_{i,j}^{2\to l}+\gamma_{\mathrm{ij}}^{l}X_{i,j}^{3\to l}+\delta_{\mathrm{ij}}^{l}X_{i,j}^{4\to l}$$ (2)

where $Y_{\mathrm{ij}}^{l}$ is the (*i*, *j*) vector of *Y*^*l*^; $\alpha_{\mathrm{ij}}^{l}$, $\beta_{\mathrm{ij}}^{l}$, $\gamma_{\mathrm{ij}}^{l}$, $\delta_{\mathrm{ij}}^{l}$ are the learnable weights from 4 different levels of feature maps to *l* layer feature maps. $\alpha_{\mathrm{ij}}^{l}$, $\beta_{\mathrm{ij}}^{l}$, $\gamma_{\mathrm{ij}}^{l}$, $\delta_{\mathrm{ij}}^{l}$ are normalized by a softmax function, where $\alpha_{\mathrm{ij}}^{l}$ is given in Eq. 3. $\beta_{\mathrm{ij}}^{l}$, $\gamma_{\mathrm{ij}}^{l}$, $\delta_{\mathrm{ij}}^{l}$ are defined similarly to $\alpha_{\mathrm{ij}}^{l}$.

$$\alpha_{\mathrm{ij}}^{l}=\frac{e^{\lambda_{\alpha_{\mathrm{ij}}}^{l}}}{e^{\lambda_{\alpha_{\mathrm{ij}}}^{l}}+e^{\lambda_{\beta_{\mathrm{ij}}}^{l}}+e^{\lambda_{\gamma_{\mathrm{ij}}}^{l}}+e^{\lambda_{\delta_{\mathrm{ij}}}^{l}}}$$ (3)

where $\lambda_{\alpha_{\mathrm{ij}}}^{l}$, $\lambda_{\beta_{\mathrm{ij}}}^{l}$, $\lambda_{\gamma_{\mathrm{ij}}}^{l}$, $\lambda_{\delta_{\mathrm{ij}}}^{l}$ are the parameters after adjusting channels by 1×1 convolution for $X_{\mathrm{ij}}^{1\to l}$, $X_{\mathrm{ij}}^{2\to l}$, $X_{\mathrm{ij}}^{3\to l}$, $X_{\mathrm{ij}}^{4\to l}$, respectively.

#### Optimization of anchor scales

**Fig. 4.**
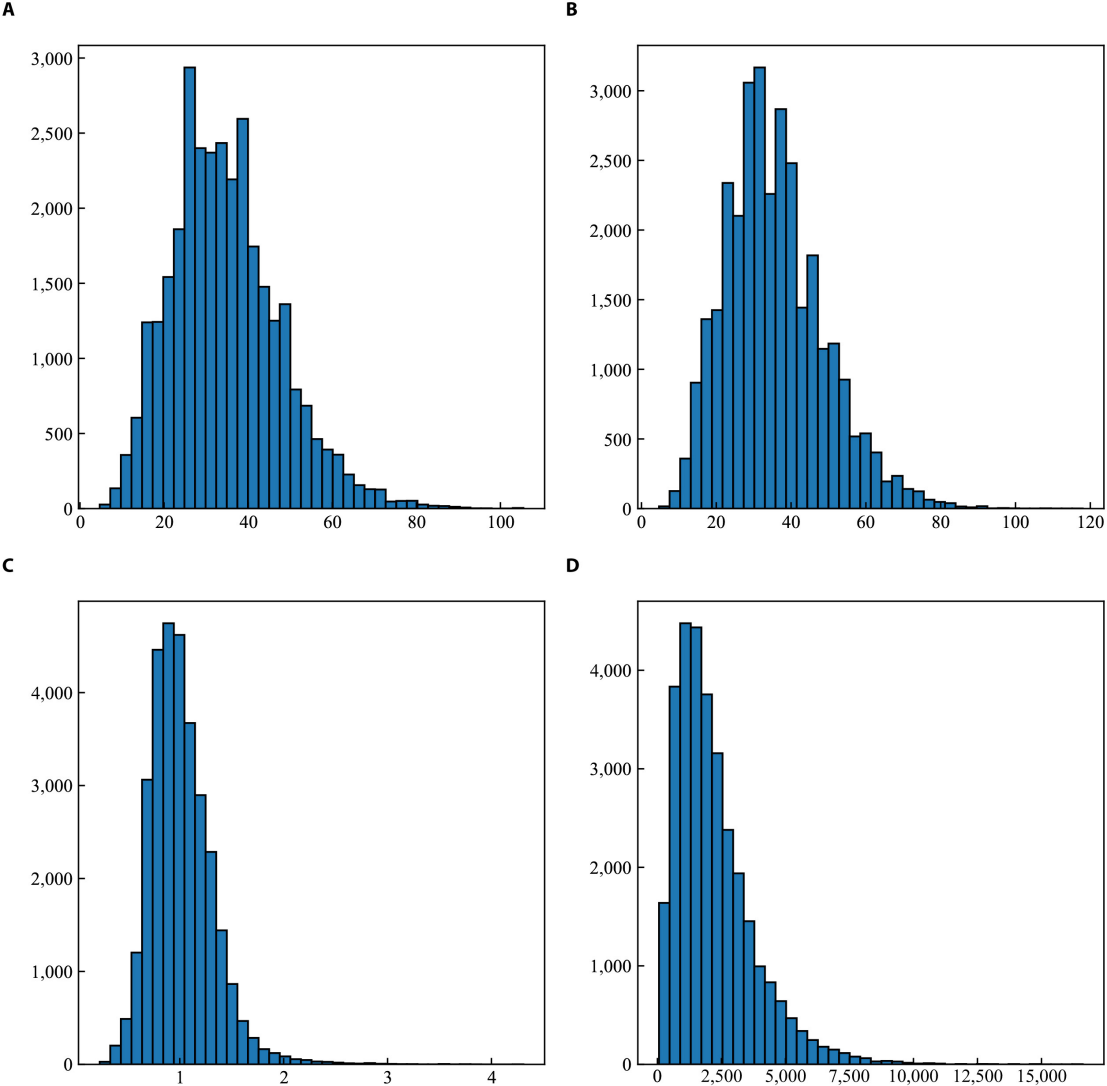
Statistics of grape berry scale. (A to D) The statistics of the number of grape berries in the dataset in terms of widths, heights, aspect ratios, and areas, respectively.

The multiscale feature layers extracted from the ASFF network are fed into RPN network. RPN extracts region of interest features from different levels of the feature pyramid according to the size of the target object. RPN is equivalent to a sliding window based on the structure of a convolutional neural network, which could generate a large number of anchor boxes with different sizes and aspect ratios. All models are built and implemented based on MMDetection. The default implementation of MMDetection [34] uses the following anchor settings: ’anchor_strides’= [4, 8, 16, 32, 64], which specifies the stride of an anchor grid. ’anchor_ratios’= [0.5, 1, 2], which is the aspect ratio per anchor box. ’anchor_scale’= [8], which is used as a multiplication factor for the strides to obtain 5 anchor scales with areas of [32 × 32, 64 × 64, 128 × 128, 256 × 256, 512 × 512]. Also, the anchor scales are fit to the objects in the Common Objects in Context (COCO) dataset. However, the sizes of grape berries in our dataset are different from objects in COCO. Considering the scaling factor of the image, the width, height, aspect ratio, and area of the actual berries input to the network by statistical analysis are shown in Fig. 4. Most of the grape berries are in the range of 5 to 100 pixels in width and height. The aspect ratio of most grape berries is in the range of 0.5 to 2. Area of grape berries is in the range of 60 to 10,000. So, in order to adjust the sizes of the berries, ’anchor_scale’ is set to [2]. The ratios are still set to [0.5, 1, 2]. The optimization of anchor scales is [8 × 8, 16 × 16, 32 × 32, 64 × 64, 128 × 128].

#### Soft-NMS

RPN filters the candidate boxes by non-maximum suppression (NMS)[35], which scores the candidate boxes with high repetition for retaining the ones with higher scores. However, according to the NMS, if the overlapping grape berry intersection over union (IoU) exceeds the set threshold value, some detection boxes containing real grape berries will be discarded, which will cause some berries to be missed. So, Soft-NMS [36] is adopted to solve this problem. Soft-NMS uses a penalty function related to the highest-scoring detection box IoU to adjust the detection box score. The larger the IoU between the detection box and the highest-scoring detection box, the greater the penalty to its detection score, while the final score is still higher than that of the nonberry area, thus ensuring that grape omission is avoided as much as possible with sufficient elimination of invalid detection frames. This paper uses the Soft-NMS with Gaussian weighting, which is calculated as

$$S_{i}=S_{i}e^{-\frac{\mathrm{IoU}{\left(M,b_{i}\right)}^{2}}{\sigma }},\forall b_{i}\notin D$$ (4)

where *S_i_* is the result of the retained candidate box, *M* is the candidate box with the highest score, *b_i_* is the candidate box that is similar to the candidate box with the highest score, IoU is the intersection ratio, and *σ* is the standard deviation.

## Results

### Experiment hardware and evaluation matrics

All the models are trained on a computer with i7 CPU and 24GB NVIDIA RTX3090. The software environment is Win10, python 3.8, and Pytorch 1.9.0. All models are built using MMDetection components. The sizes of images are set to [1333, 800], and all the images are enhanced by random horizontal and translational flipping when training. All models are trained with 2 images per batch for 12 epochs and optimized with AdamW. The initial learning rate is 0.0001 and decreases by a ratio of 0.1 after the 8th and 11th epochs, respectively. Training is performed using transfer learning, and all models are loaded with backbone weights pretrained on the Imagenet dataset. In this work, to validate the performance of all models, COCO-style [37] average precision (AP) metrics are employed, including AP0.5 and AP (AP0.5-0.95), which means the AP value when the IoU is 0.5 and 0.5 to 0.95, respectively. IoU is defined as:

$$IoU=\frac{A\cap G_{T}}{A\cup G_{T}}$$ (5)

where *A* is the predicted box and *G*_T_ is the ground truth.

APs and APm are also employed to evaluate the performance of all models. Aps and Apm represent AP for objects with an area less than 32 pixels × 32 pixels and 32 pixels × 32 pixels to 96 pixels × 96 pixels, respectively. APl is not used because APl represents objects larger than 96 pixels × 96 pixels, and according to the distribution of grape berries in Fig. 4, there are fewer berries larger than this area. A predicted box is considered as a true positive (TP) if the IoU is greater than the default threshold and the predicted class label is the same as the ground truth label. Otherwise, it is defined as a false positive (FP). Precision and recall are defined as:

$$\text{Precision}=\frac{\mathrm{TP}}{\mathrm{TP}+\mathrm{FP}}$$ (6)

$$\text{Recall}=\frac{\mathrm{TP}}{\mathrm{TP}+\mathrm{FN}}$$ (7)

$$\mathrm{AP}=\int_{0}^{1}‍\mathrm{PdR}$$ (8)

where *TP* is true positive, *FP* is false positive, and *FN* is false negative.

### Ablation study

In order to analyze the effect of every individual component (Anchor optimization, Soft-NMS, and ASFF) on our proposed model, ablation experiments are carried out on the datasets in the natural environment. Two-stage instance segmentation model configured SwinT-T as the backbone and FPN as the neck is used as the baseline. The corresponding components are added to this baseline, where the AP0.5 curve of the training process is shown in Fig. S2. The results are shown in Table 1. Anchor optimization improves the baseline by +1.9 *AP^box^*, and the other evaluation metrics are also improved compared with the baseline, which benefits from the sizes of the anchors better matching the size of grape berries. Soft-NMS brings +3.6 $AP_{0.5}^{\mathrm{box}}$ and +3.7 $AP_{0.5}^{\text{mask}}$ to the baseline, which contributes most to the final improvement. Soft-NMS also brings +6.1 $AP_{s}^{\mathrm{box}}$ to the baseline, which indicates that Soft-NMS reduces the probability of missed grape berries, especially for small grape berries. ASFF boosts the performance from 60.9 to 62.4 *AP^mask^* compared with baseline, resulting from adaptive feature fusion and enhancement. When any 2 of the 3 components are combined, the improvements over the baseline are much higher. For example, Anchor optimization + Soft-NMS could bring +7.2 $AP_{0.5}^{\mathrm{box}}$ and +6.9 $AP_{0.5}^{\text{mask}}$. While all 3 components are integrated into the baseline, the improvement is highest. The AS-SwinT is +8.6 $AP_{0.5}^{\mathrm{box}}$, +3.1 *AP^box^*, +8.9 $AP_{0.5}^{\text{mask}}$, and +2.8 *AP*^*mask*^ higher than baseline. Especially, AS-SwinT brings +13.6 $AP_{s}^{\mathrm{box}}$ and +10.4 $AP_{s}^{\text{mask}}$ to the baseline for small grape berries. This is because AS-SwinT combines the advantages of the 3 modules, preserves the characteristic information of small berries, and avoids small berries miss detection.

**Table 1.** Ablation experiment.

Anchor optimization	Soft-NMS	ASFF	$AP_{0.5}^{\mathrm{box}}$	*AP* ^*box*^	$AP_{s}^{\mathrm{box}}$	$AP_{m}^{\mathrm{box}}$	$AP_{0.5}^{\text{mask}}$	*AP* ^*mask*^	$AP_{s}^{\text{mask}}$	$AP_{m}^{\text{mask}}$
×	×	×	85.8	62.4	43.0	75.6	83.8	60.9	37.1	75.3
✓			87.7	64.4	46.1	76.1	85.8	62.1	39.5	75.6
	✓		89.4	63.2	49.1	77.3	87.5	61.2	41.0	76.3
		✓	88.8	63.5	46.9	76.9	86.8	62.4	41.1	76.8
✓			93.0	63.0	52.4	76.6	90.7	61.0	43.3	75.7
		✓	87.8	65.1	47.0	77.1	86.7	63.4	40.9	76.8
	✓	✓	89.2	64.4	49.1	78.6	87.5	62.1	41.3	77.3
✓	✓	✓	94.4	65.5	56.6	78.5	92.7	63.7	47.5	77.7

### Contrast of models with different backbone

Different backbones including ResNet50, ResNet101, ResNeXt50, ResNeXt101 [38], SwinT-T, and SwinT-S (SwinT Transformer-Small) are also used to evaluate our method. All models keep the same 3 components and configure different backbone networks; the results are shown in Table 2. It indicates that deepening backbone network layers is not effective to improve the detection and segmentation accuracy of grape berries. Even SwinT-S has a slightly reduced detection accuracy of the entire network compared to SwinT-T. Theoretically, the deeper the backbone network, the more complex local information can be extracted and higher accuracy can be achieved, but for a specific task, this may not be the case. The reason may be that as the network layer deepens, the network performance saturates, leading to a decrease in the network’s ability to learn the features of grape berries. Also, Swin Transformer brings markedly more effect on the model than ResNet and ResNeXt in different types of backbone networks. It is mainly due to the fact that Swin Transformer uses a self-attentive mechanism to accomplish long-range modeling enhancement, which gives it a strong feature extraction capability. So, it is where SwinT-T is used as the backbone network in this paper.

**Table 2.** Results of different backbones on the dataset.

Backbone	$AP_{0.5}^{\mathrm{box}}$	*AP* ^*box*^	$AP_{s}^{\mathrm{box}}$	$AP_{m}^{\mathrm{box}}$	$AP_{0.5}^{\text{mask}}$	*AP* ^*mask*^	$AP_{s}^{\text{mask}}$	$AP_{m}^{\text{mask}}$
ResNet50	93.4	64.4	55.0	77.7	91.7	62.3	45.8	76.7
ResNet101	93.3	64.2	54.5	77.5	91.6	62.0	45.4	76.1
ResNeXt50	93.6	64.6	55.6	78.1	91.9	62.7	46.1	77.0
ResNeXt101	93.6	64.8	55.2	78.0	91.9	62.4	45.9	76.7
SwinT-T	94.4	65.5	56.6	78.5	92.7	63.7	47.5	77.7
SwinT-S	93.8	64.8	56.6	78.0	92.4	63.0	47.3	77.4

### Comparisons of results with other models in artificial environment

To verify the detection and segmentation performance of our method, classical instance segmentation models including Mask R-CNN, Mask Scoring R-CNN [39], and Cascade Mask R-CNN [40] are compared with our method. SwinT-T is configured as the backbone and FPN as the neck for all models to ensure a fair comparison. The results are shown in Table 3. The proposed method could obtain 50.71M parameters and 241.79 GFLOPS, whose performance is better than Mask Scoring R-CNN and Mask R-CNN. The $AP_{0.5}^{\mathrm{box}}$ of our method is 95.2, which increased by +6.6, +6.7, and +5.5 better than Mask R-CNN, Mask Scoring R-CNN, and Cascade Mask R-CNN, respectively. The $AP_{s}^{\mathrm{box}}$ is 56.9, which increased by +3.3, +3.1, and +2.9 compared with Mask R-CNN, Mask Scoring R-CNN, and Cascade Mask R-CNN, respectively. This indicates that the detection accuracy of our method markedly exceeds other models and the small berry detection performs well. Meanwhile, Fig. S3 shows the comparative effect of different models; it could be seen that the detection and segmentation effect of AS-SwinT is substantially better than the other models. Especially for some relatively small berries, they could still be recognized by our method. It mainly depends on the ASFF, which better preserves the features of small berries through the adaptive fusion of the bottom features with the top features. Moreover, our method works better for grape berries that are covered by other berries. Because Soft-NMS is better at retaining those fruit berries overlapping each other, rather than treating them as negative samples as NMS does.

**Table 3.** Results of different models in artificial environment.

Model	Parameters (M)	GFLOPs	$AP_{0.5}^{\mathrm{box}}$	*AP* ^*box*^	$AP_{s}^{\mathrm{box}}$	$AP_{m}^{\mathrm{box}}$	$AP_{0.5}^{\text{mask}}$	*AP* ^*mask*^	$AP_{s}^{\text{mask}}$	$AP_{m}^{\text{mask}}$
Mask R-CNN	47.37	219.16	88.6	63.5	53.6	73.8	86.5	61.5	45.1	74
Mask Scoring R-CNN	63.63	219.16	88.5	63.3	53.8	74.3	86.4	60.7	45.3	74.4
Cascade Mask R-CNN	80.42	324.15	89.7	65.1	54	78.5	88.7	63.1	46.2	77.9
AS-SwinT	50.71	241.79	95.2	65.7	56.9	78.5	94.0	63.9	47.7	77.9

### Comparisons of results with other models in natural environment

**Fig. 5.**
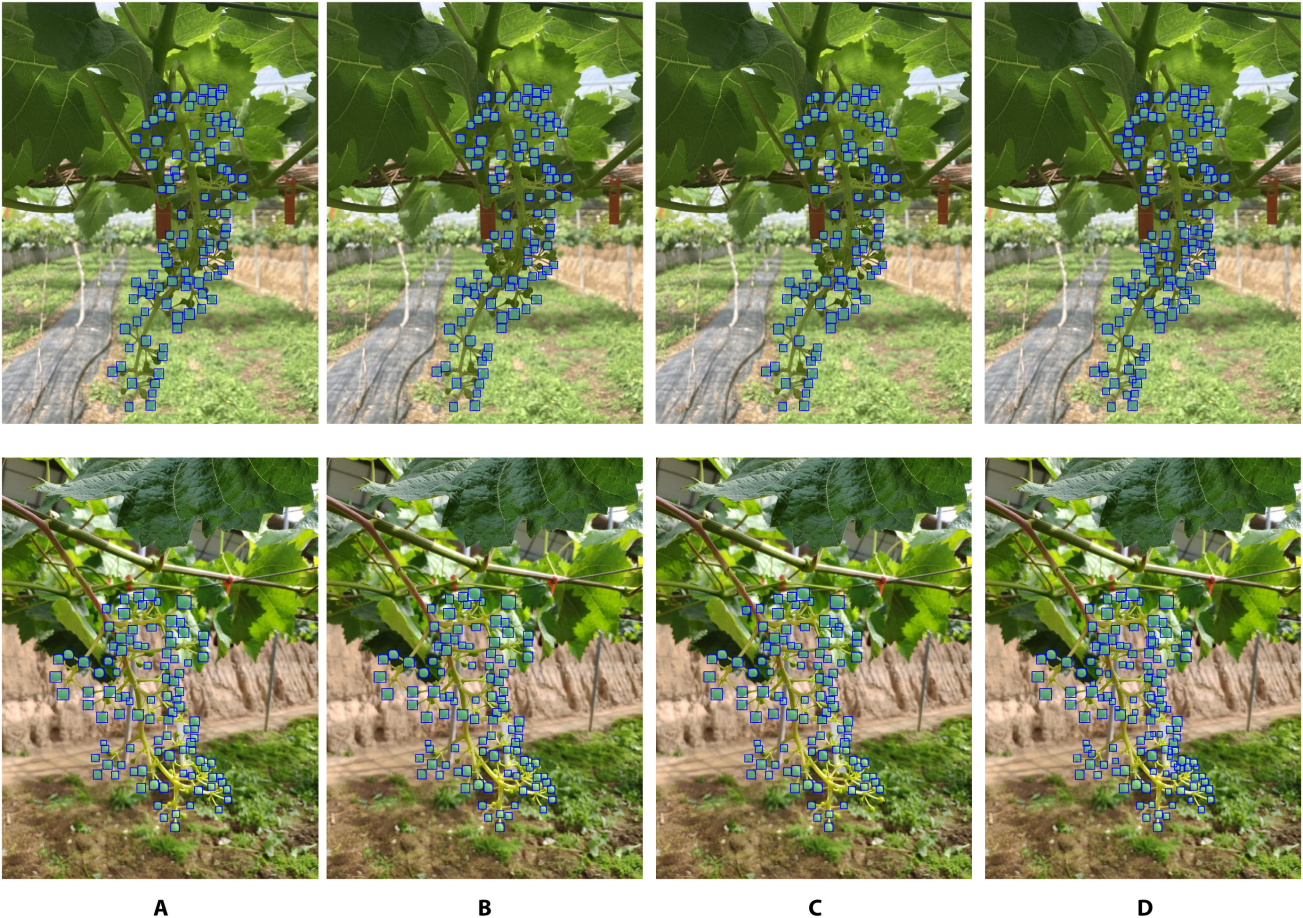
Examples of results for natural background dataset by using different models. (A to D) Samples of the detection results of Mask R-CNN, Mask Scoring R-CNN, Cascade Mask R-CNN, and AS-SwinT in natural environments, respectively.

Images in the natural background dataset are also fed into the models to evaluate the performance of our model. Especially, transfer learning for different datasets is adopted in our method. Because the features of grape berries in both datasets are the same, except for the different contexts, the artificial background dataset could be used as a transfer learning for the natural background dataset. More specifically AS-SwinT training weights under the artificial background dataset in Table 3 are reused as the initialization of the AS-SwinT model weights for the natural background dataset. Table 4 shows that AS-SwinT could get better performance compared with other models. The $AP_{0.5}^{\mathrm{box}}$ is 95, increasing by +11.1, +11.1, and +11 compared with Mask R-CNN, Mask Scoring R-CNN, and Cascade Mask R-CNN. The $AP_{s}^{\mathrm{box}}$ is 57, increasing by +9.8, +10, and +7.3 compared with Mask R-CNN, Mask Scoring R-CNN, and Cascade Mask R-CNN. What is more, through transfer learning, it is possible to obtain detection results comparable to the model performance in the artificial background dataset. Our model achieves good results in detection and segmentation compared with other models, especially when the number of grape berries is high as shown in Fig. 5.

**Table 4.** Results of different models in natural environment.

Model	$AP_{0.5}^{\mathrm{box}}$	*AP* ^*box*^	$AP_{s}^{\mathrm{box}}$	$AP_{m}^{\mathrm{box}}$	$AP_{0.5}^{\text{mask}}$	*AP* ^*mask*^	$AP_{s}^{\text{mask}}$	$AP_{m}^{\text{mask}}$
Mask R-CNN	83.9	62.9	47.2	76.2	83.9	60.9	41	77.7
Mask Scoring R-CNN	83.9	62.7	47	76.1	83.8	61	40.6	80.6
Cascade Mask R-CNN	84	63	49.7	79.3	82.9	61.8	43.7	79.1
AS-SwinT	95	65.7	57	79.2	94.3	62.8	48	79.3

The results show that AS-SwinT performs better and robustness in both datasets, as seen in Tables 3 and 4. The model achieves higher detection accuracy when tested on artificial backgrounds, likely due to the ideal conditions and more distinct features of the grape berries. In contrast, natural backgrounds can introduce environmental factors that may cause missed or incorrect detection. Table 4 shows a slight improvement in the detection and segmentation accuracy of AS-SwinT in natural background dataset compared to AS-SwinT in Table 2. Specifically, the accuracy for $AP_{0.5}^{\mathrm{box}}$, *AP^box^*, $AP_{0.5}^{\text{mask}}$, and *AP^mask^* has improved by +0.6, +0.2, +1.6, and +0.9, respectively. This benefits from the transfer learning that AS-SwinT uses the weight of the model already trained in the artificial background dataset when training in the natural background dataset. Through the use of transfer learning, AS-SwinT can use features learned in the artificial background dataset and demonstrate equivalent detection performance in the natural background dataset as it does in the artificial background dataset.

### Number estimation of berries in single bunch

AS-SwinT has the ability to predict the number of grape berries in a single bunch; however, there may be some error between the predicted and actual number. To improve the accuracy, a linear regression model can be utilized to obtain a more precise estimation. Linear regression models are often used to assess the relationship between prediction and ground truth in agriculture. So, we evaluate our method and compared it with other models by establishing a correlation between output and ground truth. The grape images are inputted into 4 models from the previous subsection to predict the number of berries, and the same linear regression model is built between the predicted and actual number of berries for each model. The loss function of this linear regression model is defined as follows.

$$L_{l}=\frac{1}{N}\sum_{i=1}^{n}{\left({\hat{y}}_{i}-y_{i}\right)}^{2}$$ (9)

where ${\hat{y}}_{i}$ is the predicted number of a single bunch; *y_i_* is the actual number of a single bunch counted manually. If the equation of this linear regression model is $\hat{y}=\mathrm{kx}+b$, then the loss function of the linear regression model is:

$$L_{l}\frac{1}{N}\sum_{i=1}^{n}{\left(\mathrm{kx}+b-y_{i}\right)}^{2}$$ (10)

where *k* and *b* are the parameters of the linear regression model. The parameters *k* and *b* of this linear regression model are found when the loss function is at a minimum.

$$k,b=\underset{k,b}{\text{argmin}}L_{l}$$ (11)

Root mean absolute error (*RMSE*) and coefficient of determination (*R*^2^) are used to reflect the goodness of fit between the 2 groups of numbers. *RMSE* and *R*^2^ are defined as:

$$\text{RMSE}=\sqrt{\frac{1}{n}\sum_{i=1}^{n}{\left({\hat{y}}_{i}-y_{i}\right)}^{2}}$$ (12)

$$R^{2}=\frac{\sum_{i=1}^{n}‍\left({\hat{y}}_{i}-\bar{y}\right)}{\sum_{i=1}^{n}‍\left(y_{i}-\bar{y}\right)}$$ (13)

where $\bar{y}$ is the actual average number.

As shown in Fig. S4, the linear regression model built between the number of berries predicted by AS-SwinT and the actual number of berries had an *R*^2^ of 0.95 for the artificial background dataset, which was the highest among the 4 models. This indicates that it has the best fitting characteristics. *RMSE* value of the model is 4.72, which is the minimum among the 4 models. This shows that it has the smallest error and the most stable fitting results in the fitting process.

**Fig. 6.**
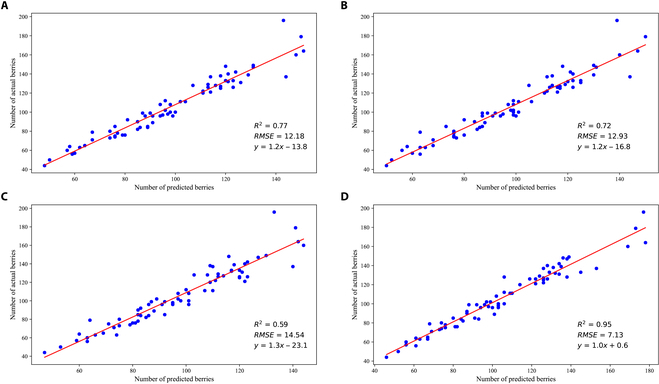
Linear regression plots of different models for natural background dataset. (A to D) Linear regression plots of Mask R-CNN, Mask Scoring R-CNN, Cascade Mask R-CNN, and AS-SwinT in natural environments, respectively.

Figure 6 shows that the *RMSE* and *R*^2^ values of AS-SwinT are 7.13 and 0.95 respectively, whose performance is better than other models obviously. In addition, our model performs comparably to the number of grape berries estimated in the natural environment as in the artificial environment, which indicates its remarkable robustness. The strong correlations of our method suggest that the number of actual berries could be estimated by the detection in RGB images, which would be highly useful to grape growers. This could benefit from the knowledge for an early decision for berry-thinning machines.

## Discussion

The method presents a robust grape berry counting based on machine vision and achieves accurate detection and segmentation. While the method has proven its utility, berries in some bunch are not successfully identified as shown in Fig. S5. This will result in inaccurate grape berry counting. Figure S5A shows that the berries heavily shaded by the main grape stem and other berries are not detected. The exposed berries are too small and similar to the patches of grass in the background, so they are mistakenly considered as negative samples by the model. Figure S5B shows that some very tiny berries are not recognized, because the features of tiny berries compared with other berries in one bunch are not remarkable, which leads to the missing detection of the model. Research would be continued in the future. Only the high-priced variety “Shine Muscat” is studied for counting. There is still a slight difference in the early stages of each grape variety. In the future, different varieties of grapes will be studied. Grape berry estimation could be affected by some conditions, such as illumination conditions and complex conditions. How to effectively eliminate the influence of these factors on the detection and segmentation results is also a work need to do in the future. Instance labeling of grape bunches requires that each berry be labeled, which is too costly. Reducing the labeling cost is the key issue of berry counting model. Key point labeling, manual virtual datasets, and semisupervised learning could provide a reference for this problem.

Berry thinning is a crucial task that will impact the market value of table grapes. The mechanization of berry thinning could meet the limitations of time constrain and lack of workers in berry thinning season. A machine vision system is the key to the berry-thinning machine, and grape berry counting could determine the number of berries removed, which is important for the vision system. In this study, an instance segmentation model AS-SwinT is designed and applied in the number estimation of grape berries for thinning in the natural environment. AS-SwinT could achieve 65.7 *AP*^*box*^, 95.0 $AP_{0.5}^{\mathrm{box}}$, 57 $AP_{s}^{\mathrm{box}}$, 62.8 *AP*^*mask*^, 94.3 $AP_{0.5}^{\text{mask}}$, 48 $AP_{s}^{\text{mask}}$ with transfer learning, which is markedly superior to Mask R-CNN, Mask Scoring R-CNN, and Cascade Mask R-CNN. In terms of the error of visual counting versus the real counts, *RMSE* and *R*^2^ of our model are 7.13 and 0.95, respectively, the linear regression results of which are equivalent to the results of the model training datasets in the artificial environment. Thus, the model has been proven robust to grapes in natural environment.

## Data Availability

The main code for our method is available at https://github.com/AWEN975/AS-SwinT.

## References

[1] Roberto SR, Borges WFS, Colombo RC, Koyama R, Hussain I, de Souza RT. Berry-cluster thinning to prevent bunch compactness of ‘BRS Vitoria’, a new black seedless grape. Sci Hortic. 2015;197:297–303.

[2] Silvestre JP, Roberto SR, Colombo RC, Azeredo Gonçalves LS, Koyama R, Shahab M, Ahmed S, de Souza RT. Bunch sizing of ‘BRS Nubia’ table grape by inflorescence management, shoot tipping and berry thinning. Sci Hortic. 2017;225:764–770.

[3] Hyun WS, Gi HK, Cheol C. Effects of plant growth regulators and floral cluster thinning on fruit quality of ‘Shine Muscat’ grape. Hortic Sci Technol. 2019;37(6):678–686.

[4] Cubero S, Diago MP, Blasco J, Tardaguila J, Prats-Montalbán JM, Ibáñez J, Tello J, Aleixos N. A new method for assessment of bunch compactness using automated image analysis: Bunch compactness assessment using image analysis. Aust J Grape Wine Res. 2015;21(1):101–109.

[5] Du W, Wang C, Zhu Y, Liu L, Liu P. Fruit stem clamping points location for table grape thinning using improved mask r-cnn. Trans Chin Soc Agric Eng. 2022;38: 169.

[6] Du W, Zhu Y, Li S, Liu P. Spikelets detection of table grape before thinning based on improved YOLOV5s and Kmeans under the complex environment. Comput Electron Agric. 2022;203: 107432.

[7] Buayai P, Yok-In K, Inoue D, Leow CS, Nishizaki H, Makino K, Mao X. End-to-end inflorescence measurement for supporting table grape trimming with augmented reality. Paper presented at: International Conference on Cyberworlds (CW); 2021 Sep 28–30; Caen, France.

[8] Buayai P, Saikaew KR, Mao X. End-to-end automatic berry counting for table grape thinning. IEEE Access. 2021;9:4829–4842.

[9] Nuske S, Wilshusen S, Achar L, Yoder SN, Singh S. Automated visual yield estimation in vineyards: Automated visual yield estimation. J Field Robotic. 2014;31(5):837–860.

[10] Aquino A, Millan B, Diago M-P, Tardaguila J. Automated early yield prediction in vineyards from on-the-go image acquisition. Comput Electron Agric. 2018;144:26–36.

[11] Pérez-Zavala R, Torres-Torriti M, Cheein FA, Troni G. A pattern recognition strategy for visual grape bunch detection in vineyards. Comput Electron Agric. 2018;151:136–149.

[12] LeCun Y, Bengio Y, Hinton G. Deep learning. Nature. 2015;521(7553):436–444. 26017442 10.1038/nature14539

[13] Palacios F, Bueno G, Salido J, Diago MP, Hernández I, Tardaguila J, Hernandez I, Tardaguila J. Automated grapevine flower detection and quantification method based on computer vision and deep learning from on-the-go imaging using a mobile sensing platform under field conditions. Comput Electron Agric. 2020;178: 105796.

[14] Coviello L, Cristoforetti M, Jurman G, Furlanello C. GBCNet: In-field grape berries counting for yield estimation by dilated CNNs. Appl Sci. 2020;10(14): 4870.

[15] Zabawa L, Kicherer A, Klingbeil L, Töpfer R, Kuhlmann H, Roscher R, Topfer R, Kuhlmann H, Roscher R. Counting of grapevine berries in images via semantic segmentation using convolutional neural networks. ISPRS J Photogramm Remote Sens, 2020;164:73–83.

[16] Deng G, Geng T, He C, Wang X, He B, Duan L. TSGYE: Two-stage grape yield estimation. *Neural information processing.* Cham (Switzerland): Springer International Publishing; 2020. Vol. 1332 of Communications in Computer and Information Science. p. 580–588.

[17] Aquino A, Diago MP, Millán B, Tardáguila J. A new methodology for estimating the grapevine-berry number per cluster using image analysis. Biosyst Eng. 2017;156:80–95.

[18] Liu S, Zeng X, Whitty M. 3DBunch: A novel iOS-smartphone application to evaluate the number of grape berries per bunch using image analysis techniques. IEEE Access. 2020;8:114663–114674.

[19] Luo L, Liu W, Lu Q, Wang J, Wen W, Yan D, Tang Y. Grape berry detection and size measurement based on edge image processing and geometric morphology. Mach Des. 2021;9(10): 233.

[20] Sun M, Xu L, Chen X, Ji Z, Zheng Y, Jia W. BFP net: Balanced feature pyramid network for small apple detection in complex orchard environment. Plant Phenomics. 2022;2022: 9892464. 36320456 10.34133/2022/9892464 PMC9595048

[21] Gai R, Chen N, Yuan H. A detection algorithm for cherry fruits based on the improved YOLO-v4 model. Neural Comput Applic. 2021;35:13895–13906.

[22] Zheng T, Jiang M, Li Y, Feng M. Research on tomato detection in natural environment based on RC-YOLOv4. Comput Electron Agric. 2022;198: 107029.

[23] Tu S, Pang J, Liu H, Zhuang N, Chen Y, Zheng C, Wan H, Xue Y. Passion fruit detection and counting based on multiple scale faster R-CNN using RGB-D images. Precis Agric. 2020;21(5):1072–1091.

[24] Shen L, Su J, Huang R, Quan W, Song Y, Fang Y, Su B. Fusing attention mechanism with mask R-CNN for instance segmentation of grape cluster in the field. Front Plant Sci. 2022;13: 934450. 35937371 10.3389/fpls.2022.934450 PMC9353517

[25] He K, Gkioxari G, Dollar P, Girshick R. Mask R-CNN. Paper presented at: Proceedings of the IEEE International Conference on Computer Vision (ICCV); 2017 Oct 22–29; Venice, Italy.

[26] Liu Z, Lin Y, Cao Y, Hu H, Wei Y, Zhang Z, Lin S, Guo B. Swin Transformer: Hierarchical vision Transformer using Shifted Windows. Paper presented at: IEEE/CVF International Conference on Computer Vision (ICCV); 2021 Oct 10–17; Montreal, Canada.

[27] Liu S, Huang D, Wang Y. Learning spatial fusion for single-shot object detection. ArXiv. 2019. 10.48550/arXiv.1911.09516

[28] Ren S, He K, Girshick R, Sun J. Faster R-CNN: Towards real-time object detection with region proposal networks. IEEE Trans Pattern Anal Mach Intell. 2017;39(6):1137–1149. 27295650 10.1109/TPAMI.2016.2577031

[29] Guo Y, Lan Y, Chen X. CST: Convolutional Swin Transformer for detecting the degree and types of plant diseases. Comput Electron Agric. 2022;202: 107407.

[30] Wang F, Rao Y, Luo Q, Jin X, Jiang Z, Zhang W, Li S. Practical cucumber leaf disease recognition using improved Swin transformer and small sample size. Comput Electron Agric. 2022;199: 107163.

[31] Bi C, Hu N, Zou Y, Zhang S, Xu S, Yu H. Development of deep learning methodology for maize seed variety recognition based on improved Swin transformer. Agronomy. 2022;12(8): 1843.

[32] He K, Zhang X, Ren S, Sun J. Deep residual learning for image recognition. Paper presented at: IEEE Conference on Computer Vision and Pattern Recognition (CVPR); 2016 Jun 27–30; Las Vegas, NV.

[33] Lin T-Y, Dollár P, Girshick R, He K, Hariharan B, Belongie S.Feature pyramid networks for object detection. Paper presented at: 2017 IEEE Conference on Computer Vision and Pattern Recognition (CVPR); 2017 Jul 21–26; Honolulu, HI.

[34] Chen K, Wang J, Pang J, Cao Y, Xiong Y, Li X, Sun S, Feng W, Liu Z, Xu X, et al. MMDetection: Open mmlab detection toolbox and benchmark. ArXiv. 2019. https://doi.org/10.48550/arXiv.1906.07155

[35] Neubeck A, Van Gool L. Efficient non-maximum suppression. Paper presented at: 18th International Conference on Pattern Recognition (ICPR’06); 2006 Aug 20–24; Hong Kong, China.

[36] Bodla N, Singh B, Chellappa R, Davis LS. Soft-NMS – Improving object detection with one line of code. ArXiv. 2017. 10.48550/arXiv.1704.04503

[37] Lin T-Y, Maire M, Belongie S, Hays J, Perona P, Ramanan D, Dollár P, Zitnick CL. Microsoft COCO: Common Objects in Context. In: Fleet D, Pajdla T, Schiele B, Tuytelaars T, editors. *Computer Vision – ECCV 2014.* Cham (Switzerland): Springer International Publishing; 2014; p. 740–755.

[38] Xie S, Girshick R, Dollár P, Tu Z, He K. Aggregated residual transformations for deep neural networks. Paper presented at: 2017 IEEE Conference on Computer Vision and Pattern Recognition (CVPR); 2017 Jul 21–26; Honolulu, HI.

[39] Huang Z, Huang L, Gong Y, Huang C, Wang X. Mask Scoring R-CNN. Paper presented at: IEEE/CVF Conference on Computer Vision and Pattern Recognition (CVPR); 2019 Jun 15–20; Long Beach, CA.

[40] Cai Z, Vasconcelos N. Cascade R-CNN: Delving into high quality object detection. Paper presented at: IEEE/CVF Conference on Computer Vision and Pattern Recognition; 2018 Jun 18–22; Salt Lake City, UT.

