# Supplementary material for: Instance Segmentation and Berry Counting of Table Grape before Thinning Based on AS-SwinT
Source: Plant Phenomics. 2023 Aug 29;5:0085. doi: 10.34133/plantphenomics.0085 (PMC10465307; doi:10.34133/plantphenomics.0085)
Supplement: Supplementary 1 — Figs. S1 to S5 Table S1 [file plantphenomics.0085.f1.doc]

Supplementary Materials


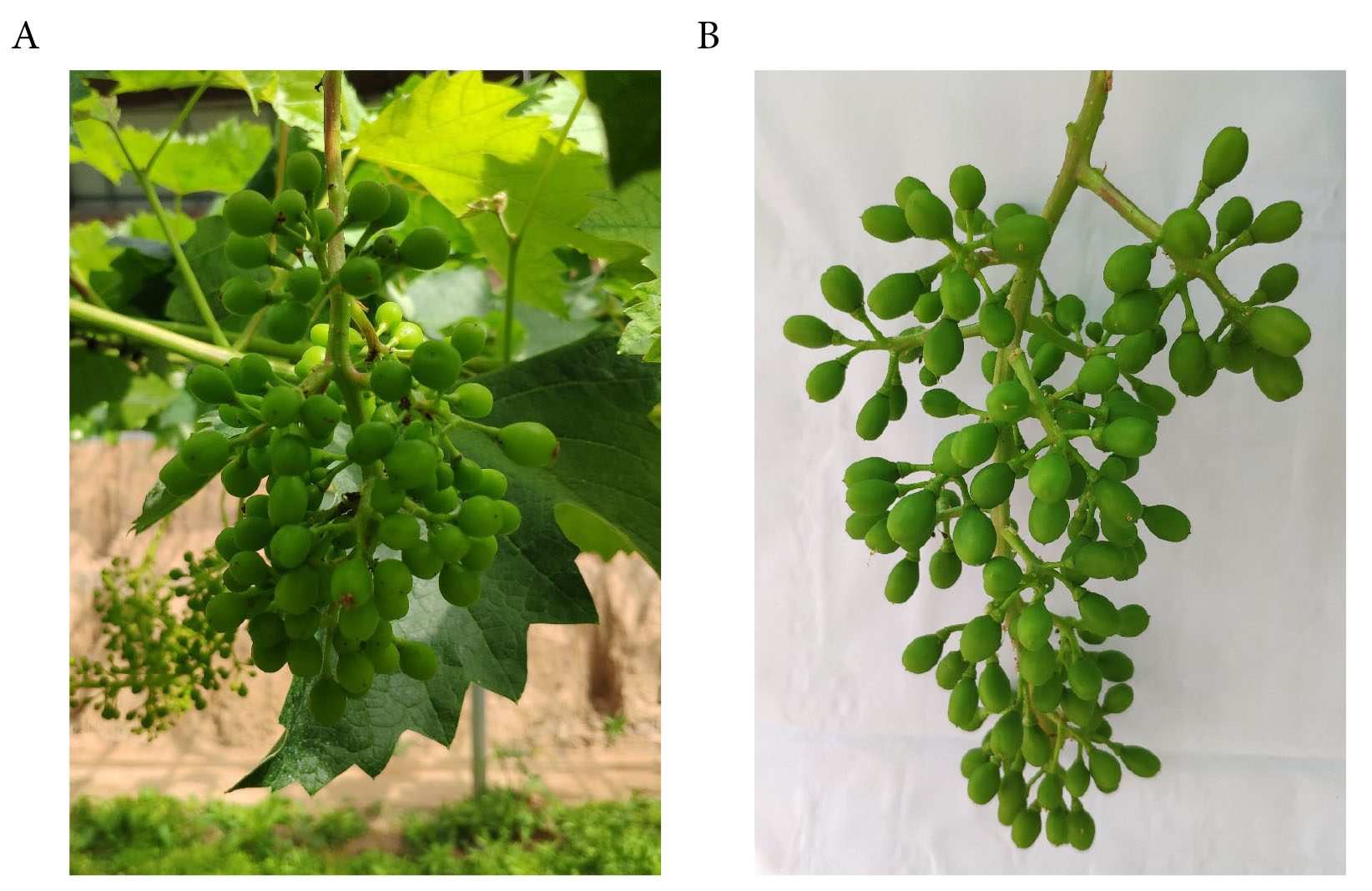


**Fig. S1** Captured images with different backgrounds. (A) An example of images in natural background. (B) An example of images in artificial background.


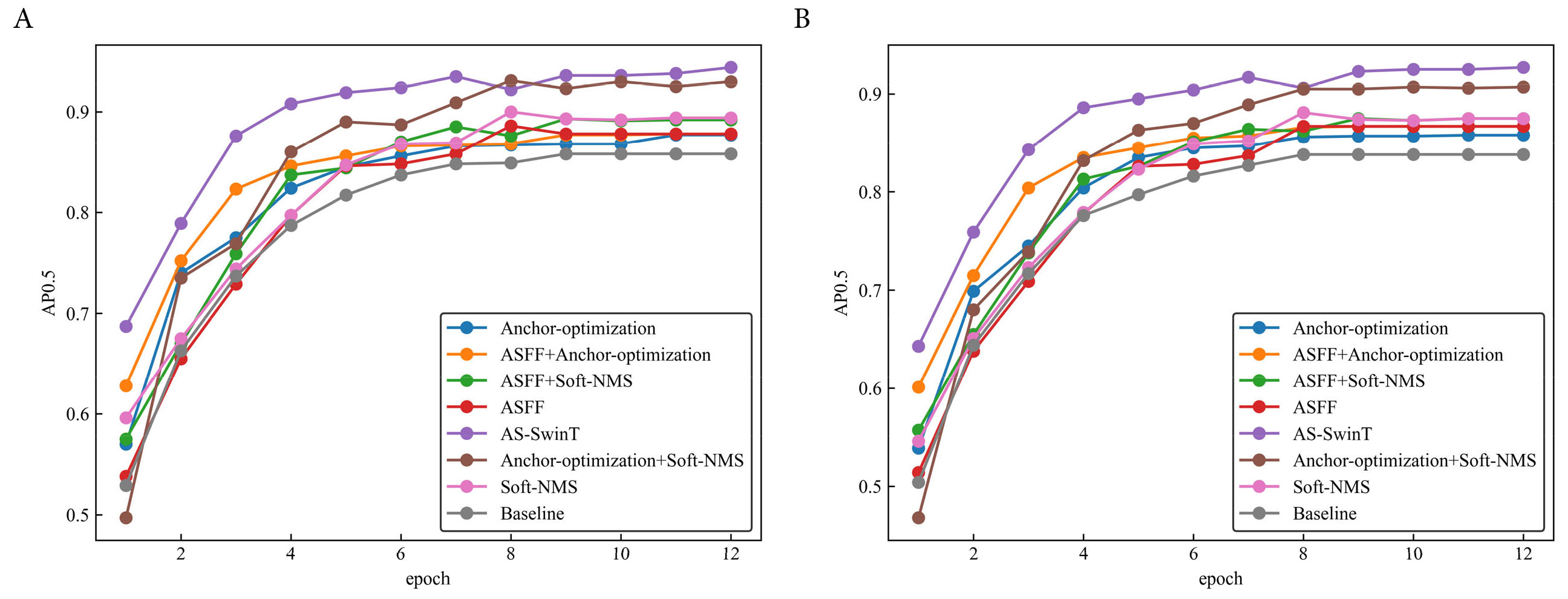


**Fig. S2** AP curves for all models. (A) and (B) are the curves of $AP_{0.5}^{box}$ and $AP_{0.5}^{mask}$ for all models in the training processes, respectively.


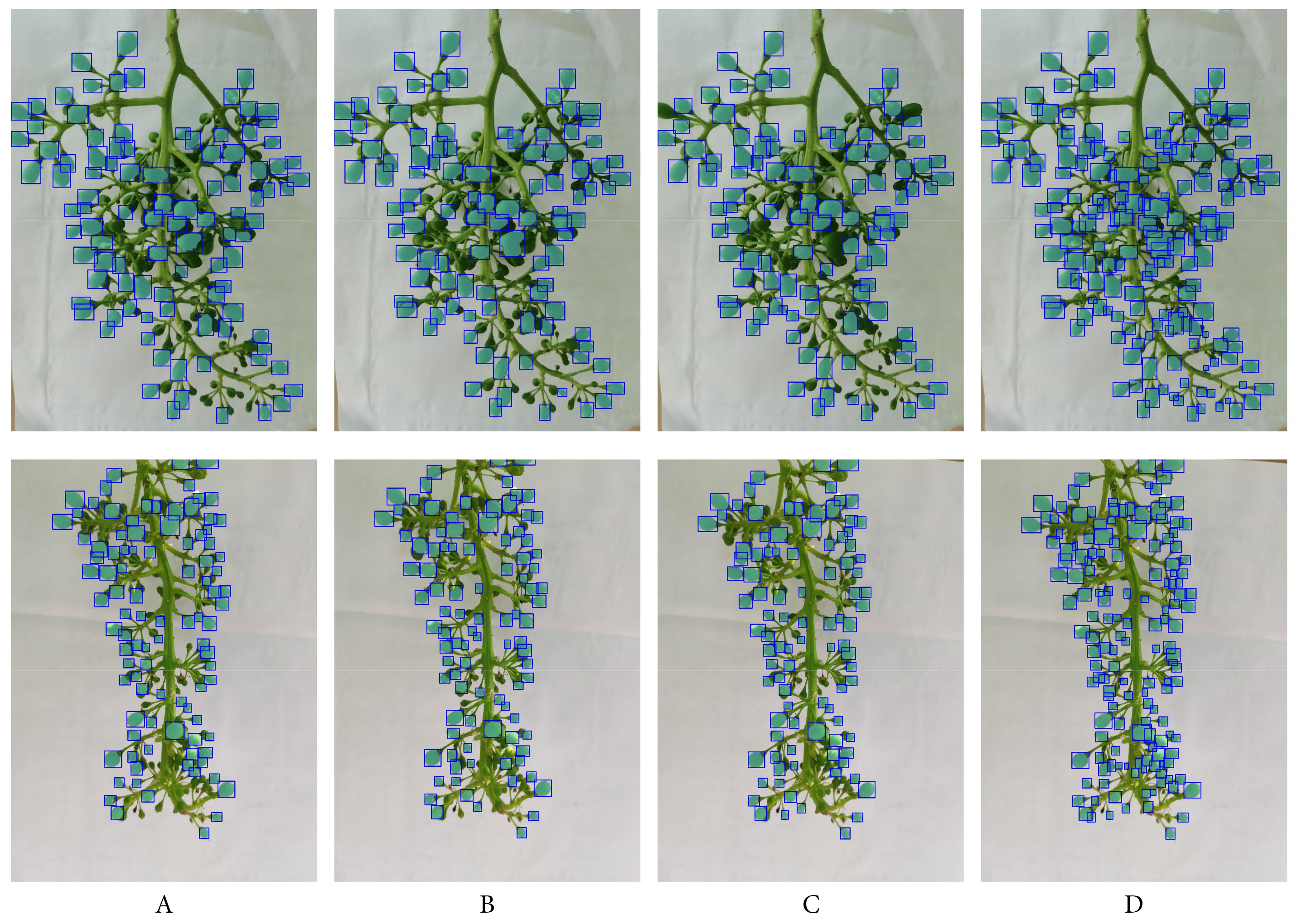


**Fig. S3** Examples of results for artificial background dataset by using different models. (A), (B), (C), and (D) are samples of the detection results of Mask R-CNN, Mask Scoring R-CNN, Cascade Mask R-CNN, and AS-SwinT in artificial environments, respectively.


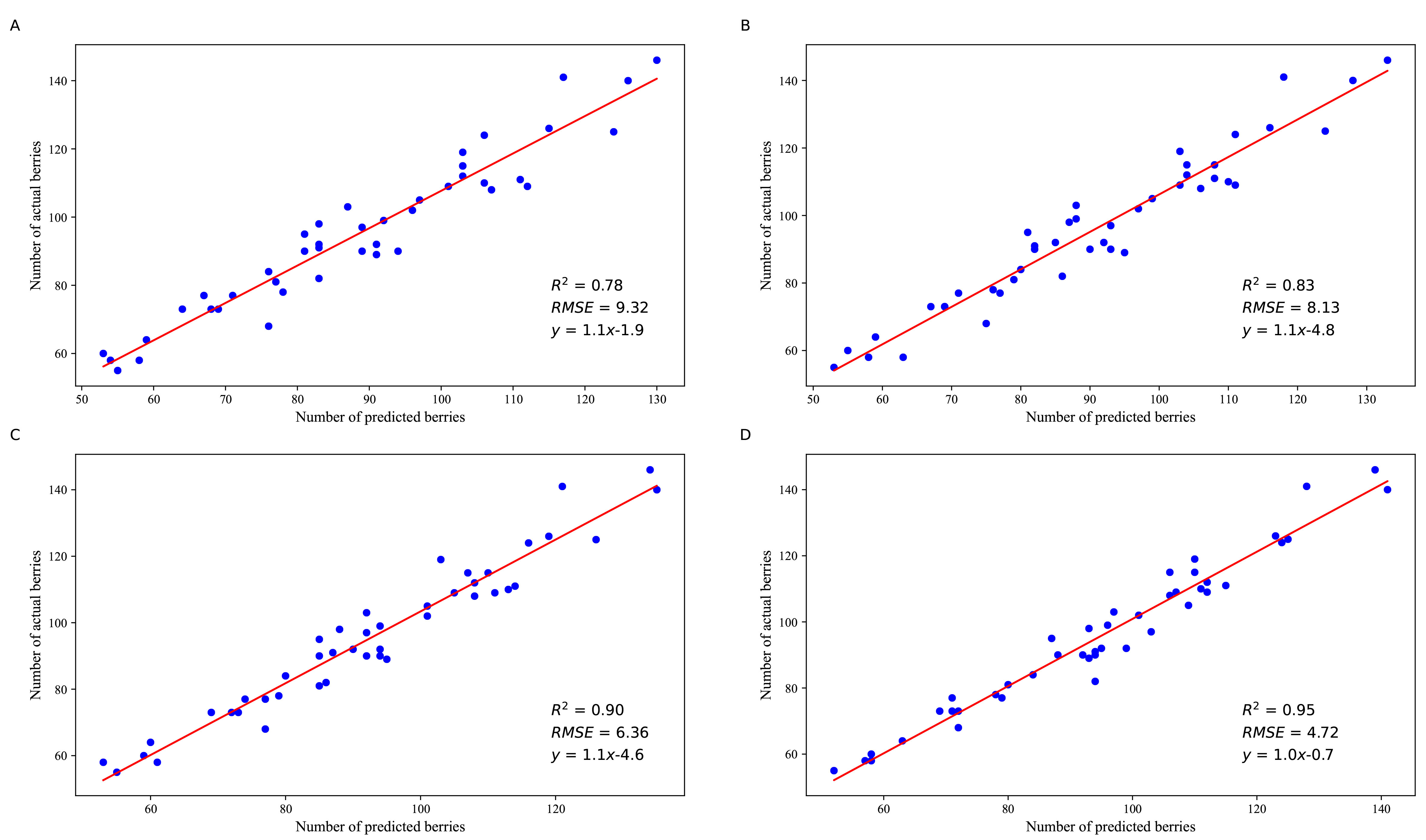


**Fig. S4** Linear regression plots of different models for artificial background dataset. (A), (B), (C), and (D) depict linear regression plots of Mask R-CNN, Mask Scoring R-CNN, Cascade Mask R-CNN, and AS-SwinT in artificial environments, respectively.


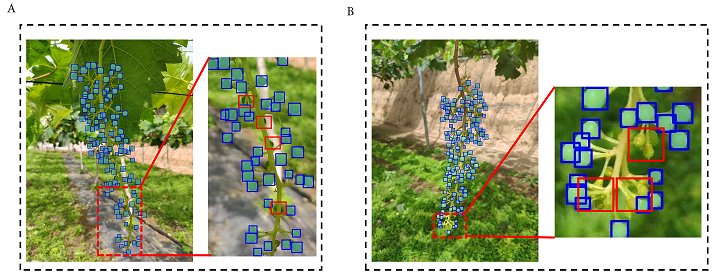


**Fig. S5** Examples of unsuccessful recognition. (A) Case 1. (B) Case 2.

**Table S1.** Dataset distribution.

| **Dataset** | **Splits** | **Total images** | **Total berries** |
| --- | --- | --- | --- |
| Natural background dataset | Training | 501 | 49960 |
|  | Validation | 144 | 14650 |
|  | Test | 72 | 7671 |
| Artificial background dataset | Training | 311 | 32160 |
|  | Validation | 89 | 8796 |
|  | Test | 44 | 4674 |
